# Personalized hemodynamic management targeting preoperative baseline cardiac index in high-risk patients having major abdominal surgery: rationale and design of the international multicenter randomized PELICAN trial

**DOI:** 10.1186/s13063-026-09657-9

**Published:** 2026-03-28

**Authors:** Moritz Flick, Eske Kvanner Aasvang, Michael Eichlseder, Adam Klimovic, Agnes S. Meidert, Christian Sylvest Meyhoff, Sebastian Roth, Moritz Steinhaus, Rune Sort, Marc Vives, Benjamin Vojnar, Sebastian Ziemann, Linda Krause, Eik Vettorazzi, Antonia Zapf, Karim Kouz, Bernd Saugel

**Affiliations:** 1 Department of Anesthesiology, Center of Anesthesiology and Intensive Care Medicine, University Medical Center Hamburg-Eppendorf, Hamburg, Germany; 2 Department of Anaesthesiology, Centre for Cancer and Organ Diseases, Rigshospitalet, Copenhagen University Hospital, Copenhagen University, Copenhagen, Denmark; 3 Division of Anaesthesiology and Intensive Care 1, Department of Anaesthesiology and Intensive Care, Medical University of Graz, Graz, Austria; 4 Department of Anesthesiology, Resuscitation and Intensive Care, Faculty of Medicine in Pilsen, Charles University, Prague, Czech Republic; 5 Department of Anaesthesiology, LMU University Hospital, LMU Munich, Munich, Germany; 6 Department of Anaesthesia and Intensive Care, Copenhagen University Hospital-Bispebjerg and Frederiksberg, Copenhagen, Denmark; 7 Department of Anesthesiology, University Hospital Düsseldorf, Heinrich Heine University Düsseldorf, Düsseldorf, Germany; 8 Department of Anesthesiology and Intensive Care, University of Lübeck and University Medical Center Schleswig-Holstein, Lübeck, Germany; 9 Department of Anaesthesiology, Copenhagen University Hospital - Amager and Hvidovre, Hvidovre, Denmark; 10 Department of Anesthesia & Critical Care, Clínica Universidad de Navarra, Pamplona, Spain; 11 Department of Anesthesiology and Intensive Care Medicine, University Hospital Giessen and Marburg, Campus Marburg and Philipps-University of Marburg, Marburg, Germany; 12 Department of Anaesthesiology, Medical Faculty, RWTH Aachen University Hospital, Aachen, Germany; 13 Institute of Medical Biometry and Epidemiology, University Medical Center Hamburg-Eppendorf, Hamburg, Germany; 14 OUTCOMES RESEARCH Consortium, Houston, TX USA

**Keywords:** Goal-directed therapy, Anesthesia, Cardiac output, Hemodynamic monitoring, Bioreactance, Perioperative medicine, Outcome, Acute kidney injury, Acute myocardial injury

## Abstract

**Background:**

Intraoperative hemodynamic management is intended to ensure adequate tissue perfusion and oxygen delivery and eventually help avoid organ injury. However, the optimal strategy for intraoperative hemodynamic management in patients having non-cardiac surgery remains unclear. We here report the protocol of a trial designed to test the hypothesis that personalized intraoperative hemodynamic management targeting preoperative baseline cardiac index reduces the incidence of a composite outcome of major postoperative complications and death within 7 days after surgery compared to routine hemodynamic management in high-risk patients having elective major abdominal surgery.

**Methods:**

The PELICAN trial is an international multicenter randomized trial in 1,128 high-risk patients having elective major abdominal surgery. The individual preoperative baseline cardiac index is determined with the patient being awake and resting in the supine position using noninvasive bioreactance. Patients are randomized to personalized hemodynamic management (intervention) or to routine hemodynamic management (control) during surgery. In patients assigned to personalized hemodynamic management, intraoperative cardiac index is maintained at least at the preoperative baseline cardiac index. In patients assigned to routine hemodynamic management, it is performed as per anesthesiologist preference (with blinded cardiac index monitoring). The primary outcome is the incidence of a composite outcome (“any event versus none”) of acute kidney injury, acute myocardial injury (including myocardial infarction), non-fatal cardiac arrest, severe infectious complications, and death within 7 days after surgery.

**Discussion:**

Our trial will determine whether personalized intraoperative hemodynamic management targeting preoperative baseline cardiac index reduces the incidence of major postoperative complications and death within 7 days in high-risk patients having elective major abdominal surgery compared to routine hemodynamic management.

**Trial registration:**

ClinicalTrials.gov Identifier NCT05648279. Registered on 5 December 2022.

**Supplementary Information:**

The online version contains supplementary material available at 10.1186/s13063-026-09657-9.

## Background

Intraoperative hemodynamic management is intended to ensure adequate tissue perfusion and oxygen delivery and eventually help avoid organ injury [1, 2]. However, the optimal strategy for intraoperative hemodynamic management in patients having non-cardiac surgery remains unclear [3–5]. Many protocols for intraoperative hemodynamic management focus on maximizing stroke volume or cardiac index during surgery. However, two recent multicenter trials reported that this approach did not improve patient-centered outcomes [6, 7].

In contrast, we consider the theory that intraoperative cardiac index targets should be personalized for each patient based on their individual preoperative baseline cardiac index. Our theory is supported by a single-center pilot trial [8] in which personalized intraoperative hemodynamic management targeting preoperative baseline cardiac index – compared to routine care – reduced a composite outcome of major postoperative complications and death within 30 days after surgery in high-risk patients having elective major abdominal surgery.

We here report the protocol of a trial designed to test the hypothesis that personalized intraoperative hemodynamic management targeting preoperative baseline cardiac index reduces the incidence of a composite outcome of acute kidney injury, acute myocardial injury, non-fatal cardiac arrest, severe infectious complications, and death within 7 days after surgery compared to routine hemodynamic management in high-risk patients having elective major abdominal surgery.

## Methods/design

### Trial design and setting

The “Personalized hemodynamic management targeting preoperative baseline cardiac index in high-risk patients having major abdominal surgery” (PELICAN) trial is an international multicenter randomized controlled single-blind clinical trial in high-risk patients having elective major abdominal surgery. The coordinating center for this trial is the University Medical Center Hamburg-Eppendorf, Hamburg, Germany. We plan to enroll 1,128 patients in 12 university medical centers (6 centers in Germany, 3 centers in Denmark, 1 center in Austria, 1 center in the Czech Republic, and 1 center in Spain). The trial is being conducted in agreement with the Declaration of Helsinki [9] and the ICH E6 R2 guidelines. The primary ethics committee for this trial is the ethics committee Hamburg (Ethikkommission der Ärztekammer Hamburg, Hamburg, Germany) which approved the trial on 15 December 2022 (registration number 2022–100955-BO-ff). Secondary approvals are obtained for each trial site by their respective ethics committee before patient recruitment starts. Written informed consent is being obtained from all participating patients. The trial was registered at ClinicalTrials.gov (NCT05648279) on 5 December 2022. We present the trial protocol following the Standard Protocol Items: Recommendations for Interventional Trials (SPIRIT) statement (Additional File 1) [10].

### Patients

Patients ≥ 45 years old who are scheduled for elective major abdominal surgery (involving visceral organs) under general anesthesia with an expected duration of surgery of ≥ 90 min AND who have at least one high-risk criterion are eligible for inclusion in the trial. The high-risk criteria are: exercise tolerance < 4 metabolic equivalents as defined by the guidelines of the American College of Cardiology/American Heart Association; renal impairment (serum creatinine ≥ 1.3 mg/dL or estimated glomerular filtration rate < 90 mL/min/1.73m^2^ within the last 6 months; coronary artery disease; chronic heart failure (New York Heart Association Functional Classification ≥ II); valvular heart disease (moderate or severe); history of stroke; peripheral arterial occlusive disease (any stage); chronic obstructive pulmonary disease (any stage) or pulmonary fibrosis (any stage); diabetes mellitus requiring oral hypoglycemic agent or insulin; immunodeficiency due to a disease (e.g., HIV, leukemia, multiple myeloma) or therapy (e.g., immunosuppressants, chemotherapy, radiation, steroids [above Cushing threshold]); liver cirrhosis (any Child–Pugh class); body mass index ≥ 30 kg/m^2^; history of smoking within two years of surgery; age ≥ 65 years; expected surgery duration ≥ 180 min; or B-type natriuretic peptide > 80 ng/L or N-terminal B-type natriuretic peptide > 200 ng/L within the last 6 months.

We do not include patients having ambulatory surgery, nephrectomy, liver or kidney transplantation surgery, or emergency surgery. We also do not include patients who had kidney, liver, heart, or lung transplantation; who have sepsis (according to current Sepsis-3 definition); who are classified American Society of Anesthesiologists physical status V or VI; who are pregnant; in whom it is impossible to perform cardiac index monitoring using the Starling Fluid Management System (Baxter, Deerfield, IL, USA); or who are participating in another clinical trial or treatment with a similar biological mechanism or primary outcome measure.

### Protocol

**Fig. 1:**
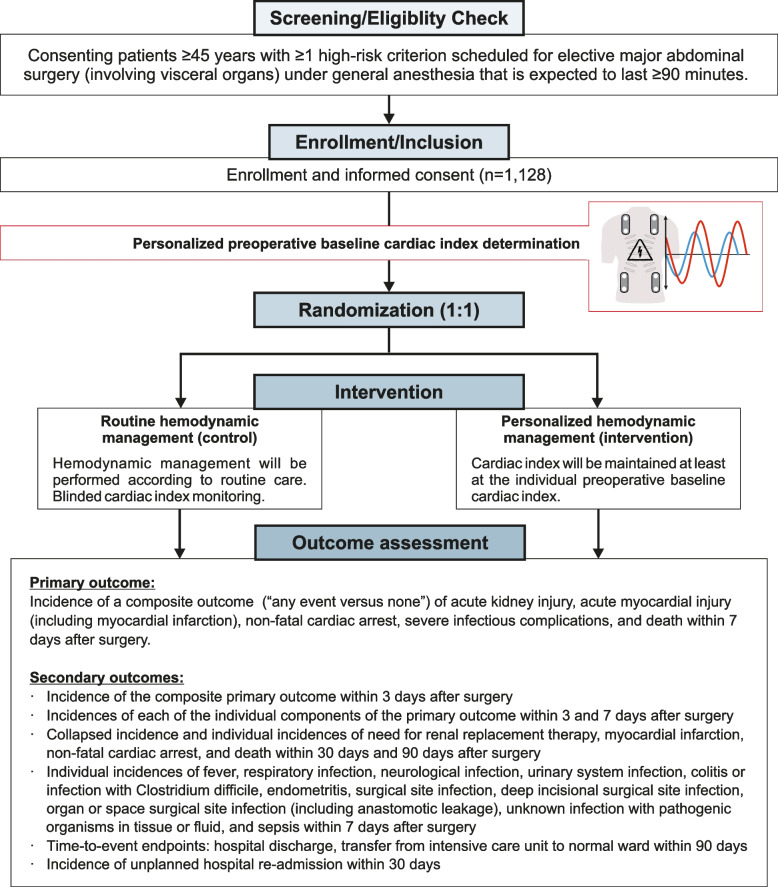
Flowchart illustrating patient screening, enrollment, randomization, trial intervention, and outcomes. Patients meeting eligibility criteria undergo preoperative baseline cardiac index measurement, followed by 1:1 randomization to either personalized hemodynamic management (intervention) or routine hemodynamic management (control). The intervention is applied intraoperatively (from start to end of surgery). The primary outcome is assessed on postoperative day 7

Figure 1 and Table 1 summarize and illustrate key aspects of the trial. Individual preoperative baseline cardiac index is determined with the patient being awake and resting in the supine position using the noninvasive bioreactance Starling Fluid Management System (usually at least one day before surgery). The individual preoperative baseline cardiac index is defined as the average value over a period of 5 min.

**Table 1:** Frequency and scope of trial visits

**Day**	**preoperative**	**perioperative**	**postoperative**						
	**-x to 0**	**0**	**1**	**2**	**3**	**4–6**	**7**	**30**	**90**
**Visits/contacts**	**I**	**II**	**III**	**IV**	**V**	**VI**	**VII**	**VIII**	**IX**
Informed consent	X								
Baseline assessment (i.e., demographic data, medical history)	X								
Preoperative baseline cardiac index measurement	X								
Blood sampling #1 (baseline values)	(X)	X							
Randomization		X							
Intraoperative cardiac index management		X							
Blood sampling #2, 3, 4, and 5			X	X	X	(X)	X*		
Primary outcome assessment							X		
Secondary outcome assessments					X		X	X	X
Serious adverse event documentation		X	X	X	X	X	X	X	X

^*^If the patient is discharged earlier than day 7, creatinine and high-sensitivity troponin concentrations will be required on the day of hospital discharge

Patients are randomized in a 1:1 ratio using central block-wise randomization with variable block length to personalized hemodynamic management (intervention) or to routine hemodynamic management (control). Randomization is stratified by center and is performed using an online system, usually on the day of surgery shortly before the start of induction of general anesthesia.

**Fig. 2:**
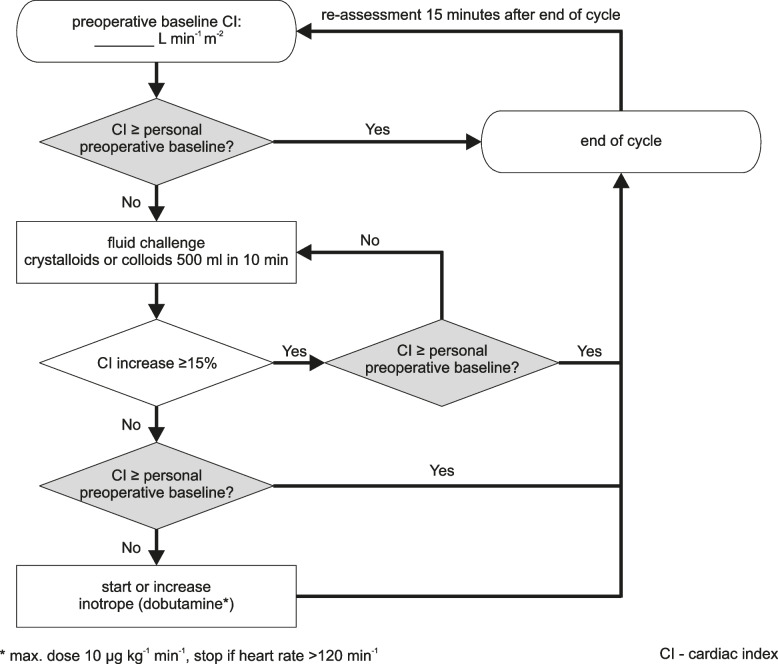
Treatment algorithm for patients assigned to personalized hemodynamic management

In patients assigned to personalized hemodynamic management, clinicians are instructed to maintain intraoperative cardiac index at least at the preoperative baseline cardiac index. In patients with a preoperative baseline cardiac index lower than 2.2 L/min/m^2^, the intraoperative cardiac index target is defined as ≥ 2.2 L/min/m^2^. Intraoperative cardiac index is measured using the Starling Fluid Management System. Patients assigned to personalized hemodynamic management receive balanced crystalloids at a baseline infusion rate of 6 mL/kg/h and additional 500 mL fluid boluses and dobutamine according to a treatment algorithm (Fig. 2). Mean arterial pressure is maintained above 65 mmHg. The trial intervention starts at the beginning of surgery and ends at the end of surgery.

In patients assigned to routine hemodynamic management, hemodynamic management is performed as per anesthesiologist preference. Intraoperative cardiac index is measured using the Starling Fluid Management System, but the clinicians are blinded to cardiac index measurements. Cardiac index monitoring can be unblinded upon request. Mean arterial pressure is maintained above 65 mmHg.

### Outcomes

The primary outcome is the incidence of a composite outcome (“any event versus none”) of acute kidney injury, acute myocardial injury (including myocardial infarction), non-fatal cardiac arrest, severe infectious complications, and death within 7 days after surgery. We will assess the following secondary outcomes:

- Incidence of the composite primary outcome within 3 days after surgery
- Incidences of each of the individual components of the primary outcome within 3 and 7 days after surgery
- Individual incidences of fever, respiratory infection, neurological infection, urinary system infection, colitis or infection with Clostridium difficile, endometritis, surgical site infection, deep incisional surgical site infection, organ or space surgical site infection (including anastomotic leakage), unknown infection with pathogenic organisms in tissue or fluid, and sepsis within 7 days after surgery
- Collapsed incidence and individual incidences of need for renal replacement therapy, myocardial infarction, non-fatal cardiac arrest, and death within 30 days and 90 days after surgery
- Time to transfer from intensive care unit to normal ward within 90 days after surgery
- Time to hospital discharge within 90 days after surgery
- Incidence of unplanned hospital re-admission within 30 days after surgery

Acute kidney injury will be defined as an increase in serum creatinine of ≥ 50% from baseline or need for renal replacement therapy [11]. Acute myocardial injury will be defined as an increase in high-sensitivity troponin according to the definition of “myocardial injury and infarction associated with non-cardiac procedures” set forth in the Fourth Universal Definition of Myocardial Infarction (2018) [12]. Serum creatinine and high-sensitivity troponin I or T (depending on the used assay in each center) will be measured before surgery (baseline; within 30 days before surgery) and on postoperative days 1, 2, 3, and 7. When patients are discharged earlier, serum creatinine and high-sensitivity troponin will be measured on the day of hospital discharge.

Severe infectious complications will be defined according to the “Standardised Endpoints in Perioperative Medicine” initiative [13] and include fever, respiratory infection, neurological infection, urinary system infection, colitis or infection with *Clostridium difficile*, endometritis, surgical site infection, deep incisional surgical site infection, organ or space surgical site infection (including anastomotic leakage), unknown infection with pathogenic organisms in tissue or fluid, and sepsis. 

Non-fatal cardiac arrest will be defined as successful resuscitation from either documented or presumed ventricular fibrillation, sustained ventricular tachycardia, asystole, or pulseless electrical activity requiring cardiopulmonary resuscitation, pharmacological therapy, or cardiac defibrillation. Death will be defined as all-cause mortality.

Data for the outcome assessment will be collected using medical records and telephone interviews on postoperative days 3, 7, 30 and 90. Serious adverse events will be documented during the trial.

### Sample size

The sample size calculation is based on the composite primary outcome. The incidence of the primary outcome was estimated to be 45% in the routine hemodynamic management group and 35% in the personalized hemodynamic management group. These estimates were based on our own pilot trial [8], which reported a 30-day composite outcome incidence of 41% in the routine care group; the OPTIMISE trial [14], which reported major complication rates of approximately 43% in control patients having major gastrointestinal surgery, and the OPTIMISE II trial protocol [15], which anticipated composite outcome rates of 45–50% in high-risk surgical patients. Our estimated 45% baseline rate is conservative and consistent with this body of evidence for high-risk patients with similar inclusion criteria. The assumed absolute risk reduction of 10% is based on an own pilot trial that investigated the effect of personalized intraoperative hemodynamic management targeting preoperative baseline cardiac index compared to routine hemodynamic management on postoperative complications and death [8].

We use a group-sequential design with one unblinded interim analysis (unblinded to an independent statistician and the members of the Data and Safety Monitoring Board (DSMB)) using the O’Brien-Fleming spending function [16]. A total sample size of 1014 patients, i.e., 507 patients per group, is required to achieve 90% power in the detection of a difference of 10% between the group incidences at a global significance level of 5% using a two-sided test of proportions without continuity correction including an unblinded interim analysis after 50% of patients are recruited. Allowing a total drop-out rate of 10% (assuming an 8% drop-out rate before randomization and 2% after randomization), a total sample size of 1,128 patients, i.e., 564 patients per group, is to be recruited. It is assumed that all patients stay within their treatment group. PASS version 2008 was used for the sample size calculation.

### Statistical analyses

Descriptive analyses will be performed to describe the patient characteristics and clinical data. The primary and secondary outcomes will be analyzed in the modified intention-to-treat population. The modified intention-to-treat population includes all patients who consented, were randomized, and had surgery. The final primary outcome analyses will be conducted using a two-sided test of proportions without continuity correction.

After a total of 564 patients had been observed for 7 days, an unblinded interim analysis was performed according to a pre-specified statistical analysis plan. It was conducted using a one-sided test of proportions without continuity correction. If the resulting p-value would have been < 0.0015 one-sided, the null hypothesis would have been rejected, and the trial would have been stopped for efficacy. However, recruitment continues. At the end of the trial, the statistical test for the primary outcome will be performed at a significance level of 0.049 two-sided.

In secondary outcome analyses, the incidences of each of the individual components of the primary outcome and the incidences of the additional secondary outcomes will be analyzed using a two-sided test of proportions without continuity correction. All time-to-event outcomes will be, due to the competing event death, assessed using Aalen-Johansen estimators and accompanied by effect estimates based on cause-specific Cox regressions.

In further analyses, the primary and secondary outcome analyses will be repeated in the per protocol population. As a sensitivity analysis, the primary outcome will be analyzed using a mixed effects logistic regression model including a fixed effect for the randomized treatment group and a random intercept for center to account for the cluster structure in the data. 

A full statistical analysis plan will be developed before any data is evaluated. Reporting will be consistent with Consolidated Standards of Reporting Trials guidelines [17]. Statistical analyses will be performed with standard statistical software such as SAS or R.

### Methods against bias

We will collect screening logs to assess the risk for selection bias in patient recruitment. To minimize bias, the following blinding procedures are implemented: (1) patients are blinded to their group assignment; (2) outcome assessors who collect and evaluate primary and secondary outcome data are blinded to group assignment; (3) data analysts performing statistical analyses are blinded to group assignment until the final analysis; (4) clinicians treating patients assigned to the routine hemodynamic management group will be blinded to patients’ preoperative resting cardiac index and intraoperative cardiac index monitoring. In these patients, intraoperative cardiac index monitoring can only be unblinded when explicit requested for clinical safety reasons; (5) randomization occurs at the last feasible moment – usually on the day of surgery shortly before induction of general anesthesia. The clinicians treating patients in the personalized hemodynamic management group cannot be blinded because they need to deliver cardiac index-guided management targeting the preoperative resting cardiac index. Clinicians treating patients in the personalized hemodynamic management group will be informed about the preoperative resting cardiac index and will have unblinded intraoperative cardiac index monitoring to guide the intervention.

### Data management and monitoring

Patient data are documented by dedicated trial personnel in an electronic case report form using a trial management software (secuTrial; secuTrial, Berlin, Germany). Data are entered in web browser input mask via an encrypted connection (HyperText Transfer Protocol Secure). Each patient receives an identification number for pseudonymized data analysis. All data entries and subsequent changes to the data, e.g., because of resolved queries, is recorded by an automated audit trail. Pseudonymized data are stored in compliance with local data protection regulations.

Trial site monitoring is carried out to ensure the protection of patients' rights and well-being, the accuracy, completeness, and verifiability of reported trial data, and adherence to the currently approved protocol and any amendments, Good Clinical Practice, and applicable regulatory requirements. Monitoring activities are performed virtual and on site and include: training trial personnel before the trial begins (site initiation visit), conducting on-site monitoring visits throughout the trial, and performing a close-out visit upon trial completion. Key aspects of the monitoring process include verifying informed consent, confirming eligibility criteria, and overseeing overall trial conduct.

### Data and Safety Monitoring Board

A DSMB consisting of three independent experts including a biostatistician oversees the trial. DSMB members met before the inclusion of the first patient, 3 months after the inclusion of the first patient, and after the interim analysis in which DSMB members reviewed the results of the interim analysis. Throughout the duration of patient recruitment, meetings of DSMB members are held every 6 months. Additional meetings may be scheduled as deemed necessary or advisable. Based on their assessment of statistical significance of the primary outcome, the data on safety, and overall progress of the trial, the DSMB members can recommend whether the trial should proceed as planned or be halted. The DSMB will operate independently of the sponsor and any conflicting interests.

### Dissemination plans

The results of this trial will be published in a peer-reviewed medical journal independent of the results. The principal investigator will prepare the final report and will have full access to all available data. Upon written request, the trial protocol, the statistical analysis plan, informed consent forms, and de-identified individual participant data can be made available. Data will be shared with researchers who provide a methodologically sound proposal for analyses that achieve aims specified in the approved proposal. Data will be provided via secure data transfer after approval of the data sharing proposal and execution of a data sharing agreement. The trial protocol and statistical analysis plan will be made publicly available immediately upon publication of the primary results via the journal's online supplementary materials. Authorship will be determined in accordance with the guidelines of the International Committee of Medical Journal Editors (http://www.icmje.org/).

## Discussion

This multicenter trial will help determine whether personalized intraoperative hemodynamic management targeting preoperative baseline cardiac index reduces postoperative complications compared to routine hemodynamic management in high-risk patients having elective major abdominal surgery. The trial will be important because – although numerous treatment algorithms for intraoperative hemodynamic management have been proposed [2, 18] – the optimal intraoperative cardiac index target value remains unknown.

Targeting predefined target values or maintaining postinduction values of stroke volume or cardiac output does not seem to improve patient outcome. In a Dutch four-center trial including 482 major abdominal surgery patients, targeting age-adapted cardiac index values (i.e. > 2.8 L/min/m^2^ for patients younger than 60 years, > 2.6 L/min/m^2^ for patients aged between 60–74 years, and > 2.4 L/min/m^2^ for patients older than 75 years) did not reduce the average number of major complications within a month after surgery [19]. In a multicenter trial including 380 high-risk patients having major abdominal surgery, maintaining optimized postinduction cardiac index throughout surgery and for the first 8 postoperative hours did not reduce the incidence of a composite outcome of complications within 28 days after surgery compared to routine care [6]. Similarly, maximizing and maintaining postinduction stroke volume throughout surgery and for the first four postoperative hours did not reduce the incidence of postoperative infections within 30 days of randomization in patients having major gastrointestinal surgery compared to routine care in the 2498-patient OPTIMISE II trial [7]. In contrast to these previous trials, we use personalized intraoperative cardiac index targets based on preoperative resting cardiac index – which varied between 2 to 5 L/min/m^2^ in patients presenting for elective major abdominal surgery in our pilot trial [8].

We are including high-risk patients (≥45 years old with at least one high-risk criterion) having elective major abdominal surgery to ensure that the studied population is broad and representative of the patient population at risk of postoperative complications [20]. Participating trial centers are large university medical centers with high volumes of high-risk patients having different types of major abdominal surgery procedures to allow efficient recruitment.

We will assess preoperative baseline and intraoperative cardiac index using the bioreactance method (Starling Fluid Management system). Bioreactance allows operator independent noninvasive continuous cardiac index monitoring using adhesive skin sensors. Noninvasiveness is a key aspect for the preoperative baseline cardiac index assessment to ensure patient comfort and safety. We chose the bioreactance method also for intraoperative cardiac index monitoring to guarantee comparability between preoperative and intraoperative cardiac index values. The bioreactance method has been evaluated and validated in different settings [21, 22]. One may argue that minimally invasive pulse wave analysis based on the blood pressure waveforms recorded with an arterial catheter – the most commonly used method to monitor cardiac index in non-cardiac surgery [23] – would have been a preferable alternative. However, the measurement performance of available minimally invasive pulse wave analysis monitors is similar to that of monitors using bioreactance [24].

Unlike other treatment algorithms, ours includes only cardiac index and no additional hemodynamic variables. To maintain patients' individualized cardiac index targets, the algorithm first employs fluid administration until the patient is no longer fluid responsive, and only then introduces dobutamine infusion. For fluid administration, clinicians may select either colloids or crystalloids at their discretion. Dobutamine infusion rates can be titrated according to specific patient responses.

This pragmatic approach simplifies interpretation and implementation. While supplementary hemodynamic variables might provide additional information about the hemodynamic status, their application is often limited in high-risk abdominal surgery – e.g., due to low tidal volumes or capnoperitoneum [25].

The selected outcomes are perfusion-related and clinically important [26, 27]. Using standardized outcomes recommended by the “Standardised Endpoints in Perioperative Medicine” initiative will allow meaningful comparisons with other trials [28]. We chose to only consider complications within the first 7 postoperative days for the primary outcome to reduce the risk of confounding by postoperative events rather than detecting the effect of intraoperative hemodynamic management. Secondary outcomes include the analysis of postoperative complications within 30 and 90 days after surgery to account for severe longer-term complications. Ultimately, our trial will determine whether personalization of intraoperative cardiac index improves postoperative outcomes.

In summary, we will perform a randomized trial to test the hypothesis that personalized intraoperative hemodynamic management targeting preoperative baseline cardiac index reduces the incidence of a composite outcome of acute kidney injury, acute myocardial injury, non-fatal cardiac arrest, severe infectious complications, and death within 7 days after surgery compared to routine hemodynamic management in high-risk patients having elective major abdominal surgery.

### Trials status

Patient recruitment for this trial started in October 2023 and is estimated to be completed in September 2025. This article is based on the most recent version of the trial protocol (i.e., version 1.5, 11 July 2025).

## Supplementary Information

Supplementary Material 1. SPIRIT checklist.

## Data Availability

Not applicable.

## Abbreviations

DSMB: Data and Safety Monitoring Board
SPIRIT: Standard Protocol Items: Recommendations for Interventional Trials
